# Topical Skullcapflavone II attenuates atopic dermatitis in a mouse model by directly inhibiting associated cytokines in different cell types

**DOI:** 10.3389/fimmu.2022.1064515

**Published:** 2022-12-20

**Authors:** Youngae Lee, Jang-Hee Oh, Na Li, Hyun-Jae Jang, Kyung-Seop Ahn, Sei-Ryang Oh, Dong Hun Lee, Jin Ho Chung

**Affiliations:** ^1^ Department of Dermatology, Seoul National University College of Medicine, Seoul, Republic of Korea; ^2^ Institute of Human-Environment Interface Biology, Medical Research Center, Seoul National University, Seoul, Republic of Korea; ^3^ Laboratory of Cutaneous Aging Research, Biomedical Research Institute, Seoul National University Hospital, Seoul, Republic of Korea; ^4^ Department of Biomedical Sciences, Seoul National University Graduate School, Seoul, Republic of Korea; ^5^ Natural Medicine Research Center, Korea Research Institute of Bioscience and Biotechnology, Cheong-ju, Chungcheongbuk-do, Republic of Korea

**Keywords:** Skullcapflavone II, atopic dermatitis, pruritus, Th2 cytokines, IgE

## Abstract

Skullcapflavone II (SFII), a flavonoid derived from *Scutellaria baicalensis*, is an anticancer agent. We aimed to validate SFII for atopic dermatitis (AD) therapy by demonstrating the anti-inflammatory effects of SFII in an AD mouse model produced by the topical application of the vitamin D3 analog MC903. We showed that topical treatment with SFII significantly suppressed MC903-induced serum IgE levels compared with topical hydrocortisone (HC) treatment. Topical SFII also prevents MC903-induced pruritus, skin hyperplasia, and inflammatory immune cell infiltration into lesional skin comparable to topical HC. In addition, MC903-induced immune cell chemoattractants and AD-associated cytokine production in skin lesions were effectively suppressed by topical SFII. The production of MC903-induced effector cytokines influencing T helper (Th)2 and Th17 polarization in lesioned skin is significantly inhibited by topical SFII. Furthermore, we showed that SFII can directly inhibit the production of AD-associated cytokines by human primary keratinocytes, mouse bone marrow-derived cells (BMDCs), and mouse CD4^+^ T cells *in vitro*. Lastly, we demonstrated that topical SFII more effectively suppressed serum IgE levels, the production of IL-4 and thymic stromal lymphopoietin (TSLP), and infiltration of CD4^+^ T cells and Gr-1^+^ cells (neutrophils) into lesion skin compared to topical baicalein (a flavonoid derived from *Scutellaria baicalensis*), which has anti-inflammatory effects. Taken together, our findings suggest that SFII may have promising therapeutic potential for this complex disease *via* the regulation of multiple AD-associated targets.

## Introduction

Atopic dermatitis (AD) is a common chronic inflammatory skin disease characterized by complex pathogenesis and a wide spectrum of clinical phenotypes presenting with underlying skin barrier dysfunction, immune dysregulation, and pruritus (1–3). It has been reported that epidermal barrier disruption and activation of epidermal dendritic cells (DCs) lead to T helper 2 (Th2)-type immune responses (IL-4 and IL-13), which are mostly activated in patients with AD. In addition, strong activation of Th1 (IFN-γ) and Th17 (IL-17A, IL-6, and S100A8) immune responses have been reported in Asian patients with AD (4–6). A previous study has shown that common AD transcriptomes such as *S100A8/A9/A12* and *CXCL1* are increased in the lesioned skin of patients with moderate-to-severe AD by RNA-sequencing and microarray analyses (7). The secretion of chemokines (e.g., CXCL1,2,5 and CCLs) by keratinocytes is involved in the infiltration of immune cells such as T cells, mast cells, basophils, and neutrophils (8). Lesional skin infiltration by these immune cells has been shown to further promote AD-associated inflammatory cascades, and itching, as well as epidermal and dermal hyperplasia (9–11). Thus, AD is a complex and heterogeneous inflammatory skin disease (12–14). Numerous therapeutic drugs for the treatment of AD have been developed and are currently under clinical investigation to identify novel therapeutic targets for more effective long-term control of complex AD (1).

The calcipotriol (MC903)-induced AD mouse model is well established and is widely used for research on the pathophysiology of human AD (15, 16). The MC903 model is ideal for experimental approaches because of its highly reproducible phenotypes that closely resemble human AD and its ability to induce the development of lesions and itching (17). The underlying cellular and molecular mechanism of MC903-induced AD-like inflammation are well known. MC903 initially induces the secretion of an alarmin, thymic stromal lymphopoietin (TSLP), from keratinocytes through a cell autonomous event dependent on retinoid X receptor/vitamin D receptor (15), which then induces orchestrated DC-T cells-basophils-T cells cascade-derived Th2-type immune responses by stimulating IL-4 and IL-13 production by CD4^+^ T cells in skin-draining lymph nodes (15, 18). Therefore, TSLP is considered a key initiation factor for exacerbated Th2 responses and is thought to be one of the therapeutic targets in AD. A recent study has shown that TSLP directly promotes pathogenic Th2 cell differentiation (19). TLSP also mediates the expansion of functionally distinct basophils, which promote Th2-type immune responses (20). In addition, MC903-induced neutrophil infiltration into the lesional skin causes enhanced expression of itching-associated molecules and inflammatory cytokines, leading to chronic itch and inflammation (9). Moreover, IL-4 and IL-13 are crucially involved in the direct activation of sensory neurons in humans and mice, leading to chronic itching through the activation of their neuronal receptors (21). It has also been reported that MC903 induces high concentrations of serum immunoglobulin E (IgE), as observed in extrinsic AD pathology (22, 23). IL-4 produced by activated CD4^+^ T cells and mast cells also promotes the production of serum IgE by activating B cells and plasma cells, which mediate hypersensitivity reactions in allergic diseases such as AD and asthma (24–26).

We have previously shown that skullcapflavone II (SFII), a flavonoid derived from *Scutellaria baicalensis*, inhibits the production of thymus and activation-regulated chemokine (TARC/CCL17) and macrophage-derived chemokine (MDC) in HaCaT cells, following stimulation with TNF-α and IFN-γ (27). It has been reported that plasma levels of TARC and MDC are elevated in patients with AD, which is strongly correlated with disease severity (28, 29). The flavonoid, baicalein, is another active component derived from *Scutellaria baicalensis* and has been reported to have anti-inflammatory effects in atopic dermatitis in NC/Nga mice (30). Thus, these results prompted us to explore the novel possibility of using SFII as a safe and effective therapeutic agent against AD.

In this study, we clarified the therapeutic effects of SFII in AD using an MC903-induced AD-like mouse model. In addition, we demonstrated the direct inhibitory effects of SFII on the production of AD-associated cytokines by human primary keratinocytes and mouse immune cells *in vitro*.

## Materials and methods

### Antibodies and reagents

Antibodies against p-STAT1 (sc-7988) and t-STAT1 (sc-592) were purchased from Santa Cruz Biotechnology Inc. (Santa Cruz, CA, USA). Antibodies against p-p65 (CST3031S), t-p65 (CST8242S), p-ERK1/2 (CST9101S), t-ERK1/2 (CST9102S), p-JNK (CST9251S), t-JNK (CST9252S), p-p38 (CST9211S), and t-p38 (CST9212S) were purchased from Cell Signaling Technology Inc. (Danvers, MA, USA). Horseradish peroxidase-conjugated polyclonal secondary antibodies against mouse, rabbit, or goat IgG (GeneTex, Inc., Irvine, CA, USA) were also purchased. Antibodies against mouse CD4 (Clone 4SM95) and mouse Gr-1(Clone RB6-8C5) were purchased from Invitrogen (Carlsbad, CA, USA) and R&D Systems (Minneapolis, MN, USA), respectively. The mouse eosinophil antibody (Clone BMK-13) was purchased from Novus Biologicals (Littleton, CO, USA). Inhibitors SB203580 (p38 inhibitor), SP600125 (JNK inhibitor), U0126 (MEK1 inhibitor), and MC903 were purchased from Tocris (Bristol, UK), and InSolution™ JAK inhibitor I and BAY 11-7082 (NF-κB inhibitor) were purchased from Calbiochem (San Diego, CA, USA). SFII was obtained from the Korea Research Institute of Bioscience and Biotechnology (KRIBB, Daejeon, South Korea) (31). Baicalein and hydrocortisone were purchased from Sigma-Aldrich (St. Louis, MO, USA). Poly(I:C), peptidoglycan (PGN), and lipopolysaccharide (LPS) were purchased from *In vivo* Gen (San Diego, CA, USA).

### Mice

Female BALB/c mice, 6–8 weeks old, were purchased from Koatech (Pyeongtaek, Korea), and maintained under specific pathogen-free conditions. All animal experiments were approved by the Seoul National University Hospital Institutional Animal Care and Use Committee (No. 20-0112-S1A0) and were performed in accordance with the Guidelines for the Care and Use of Laboratory Animals at Seoul National University Hospital.

### MC903-induced AD-like skin inflammation in mice

In the MC903-induced AD-like model, 1.0 nmol of MC903 in 25 μl ethanol were topically applied on both ears for 7 days. After 2 h, both ears were topically treated with 25 μl of SFII, baicalein, HC, or ethanol (vehicle control). After 7 consecutive days, the ears were topically treated with SFII, baicalein, HC, or ethanol (vehicle control) daily without MC903. MC903 or compounds were dissolved in 100% ethanol. The ear thickness was measured daily using a digital caliper (Mitutoyo Corp., Tokyo, Japan). At the end of the experiment on day 13, the ear skin was snap-frozen in liquid nitrogen for RNA and protein isolation, and the ear was embedded in OCT compounds to prepare frozen sections. The number of scratch bouts were monitored and quantified for 30 min. One bout of scratching was defined as an episode in which a mouse lifted its paw and scratched continuously for any length of time, until the paw was returned to the floor (32). Scratching counts were assessed with the investigator blinded to the groups.

### Histology, immunofluorescence analysis, and toluidine blue staining

Ear skin was fixed in 4% paraformaldehyde at 4°C overnight, paraffin-embedded, sectioned into 4 µm, and stained with hematoxylin and eosin (H&E). Images were acquired using a microscope. Epidermal and dermal thicknesses were measured using ImageJ software (NIH). For immunofluorescence, ear tissue sections were blocked in pre-blocking solution (GBI Labs, Bothell, WA, USA) and stained with the indicated primary antibodies at 4°C overnight. After washing, the sections were incubated with Alexa Fluor 488- or Alexa Fluor 594-conjugated secondary antibodies at 25°C for 1 h and stained with 4′-6-diamidino-2-phenylindole dihydrochloride (Thermo Fisher Scientific, Waltham, MA, USA) at 25°C for 5 min. Images were acquired using a confocal microscope (Leica STED CW; Leica Microsystems, Mannheim, Germany). For the detection of mast cells, ear sections were stained with 0.5% toluidine blue at room temperature for 15 min and then washed three times in PBS. Images were acquired using a microscope. The number of immune cells was counted every five fields (40× objective), and the average was calculated.

### Quantitative PCR

Total RNA from the ear skin was isolated using RNAiso Plus (Takara Bio Inc., Shiga, Japan) according to the manufacturer’s instructions. Then, 1.0 μg of total RNA was used for cDNA synthesis using the First Strand cDNA Synthesis Kit (Thermo Fisher Scientific). Quantitative RT-PCR was carried out using SYBR Premix (Bioneer, Daejeon, Korea) and quantitatively measured with an ABI Real-Time PCR instrument (ABI, Indianapolis, IN, USA). Each sample was analyzed in duplicate and the relative expression of mRNA was normalized to that of the housekeeping gene 36B4.

### ELISA

Serum IgE levels were measured using ELISA according to the manufacturer’s protocol (BioLegend, San Diego, CA, USA). The level of *in vivo* cytokine production in mice was measured using ELISA according to the manufacturer’s protocol (BioLegend). Additionally, the level of human TSLP in the cell culture supernatants was determined by ELISA according to the manufacturer’s protocol (R&D Systems).

### Western blot analysis

Human primary keratinocytes were pretreated with SFII for 30 min, then stimulated with 10 μg/ml poly(I:C) for 3 h. Cells were washed with PBS, lysed with 1×TNE buffer (20 mM Tris-HCl, 150 mM NaCl, 1 mM EDTA, and 1% NP-40) containing complete mini protease inhibitor cocktails (Roche Applied Science, Indianapolis, IN, USA) and phosphatase inhibitor cocktails (Sigma-Aldrich), and heated at 95°C for 5 min. Equal amounts of cell lysate were separated on a 10% SDS-polyacrylamide gel by electrophoresis and transferred onto a nitrocellulose membrane (GE Healthcare, Chicago, IL, USA). Equal protein transfer was confirmed by Ponceau S staining (ELPIS Biotech, Daejeon, South Korea). The membranes were blocked with 5% skim milk in Tris-buffered saline with 0.1% Tween^®^ 20 (TBS-T) and probed overnight with the indicated primary antibodies at 4°C. After washing with TBS-T, the membranes were probed with HRP-conjugated secondary antibodies for 1 h at 25°C and visualized using WestGlow™ PICO PLUS or FEMTO chemiluminescent substrates (Biomax Co., Ltd., Seoul, Korea) using a CCD imaging system (Amersham Imager 680, GE Healthcare). Only the data with unsaturated signals were used in the analysis.

### Human primary keratinocytes

Human epidermal keratinocytes were isolated as described previously (33). Human epidermal keratinocytes were cultured in a keratinocyte growth medium (KBM™ Gold™ Basal Medium; Lonza, Basel, Switzerland) supplemented with KGM™ Gold™ SingleQuots™ supplements (Lonza). Human epidermal keratinocytes were used at the third or fourth passage. Human primary keratinocytes were isolated from the epidermis of juvenile foreskin from two donors.

### Generation of bone marrow cell-derived DCs

Bone marrow (BM) cells were collected from the femurs and tibias of the BALB/c mice. BM-derived dendritic cells (BMDCs) were generated by culturing BM cells from mice in complete RPMI 1640 supplemented with 20 ng/ml recombinant murine GM-CSF (PeproTech, Rocky Hill, NJ) (34, 35). Nonadherent cells were harvested on day 6 and stimulated for 24 h with TLR agonists, PGN or LPS (*In vivo*Gen), in the presence of vehicle or SFII. BMDC culture supernatants were collected and analyzed for cytokine production by ELISA.

### Isolation and activation of mouse CD4^+^ T cells

Mouse CD4^+^ T cells from the spleen were isolated using magnetic activated cell sorting (MACS) LS columns, following the manufacturer’s instructions (Miltenyi Biotec, Bergisch Gladbach, North Rhine-Westphalia, Germany). For mouse T cell proliferation assays, purified CD4^+^ T cells were labeled with 1 mM CFSE (Invitrogen), according to the manufacturer’s protocol. T cells (2 × 10^5^) were stimulated with plate bound anti-CD3ϵ (5 µg/ml, clone 145-2C11) and soluble anti-CD28 (2 µg/ml, clone 37.51) antibodies for 72 h, in the presence of vehicle or SFII (36). The intensity of CFSE was analyzed using a FACSCanto II flow cytometer (BD Biosciences, San Jose, CA, USA).

### Statistical analysis

Statistical analyses were performed using Prism 9.0 software (GraphPad Software, La Jolla, CA, USA). Dunnett’s multiple range test was used to compare differences between two groups. Two-way ANOVA was used to compare differences between multiple groups. All graphs are presented as the mean ± standard error. The threshold for statistical significance was set at *P <*0.05.

## Results

### Topical SFII attenuates MC903-induced AD-like skin inflammation

**Figure 1:**
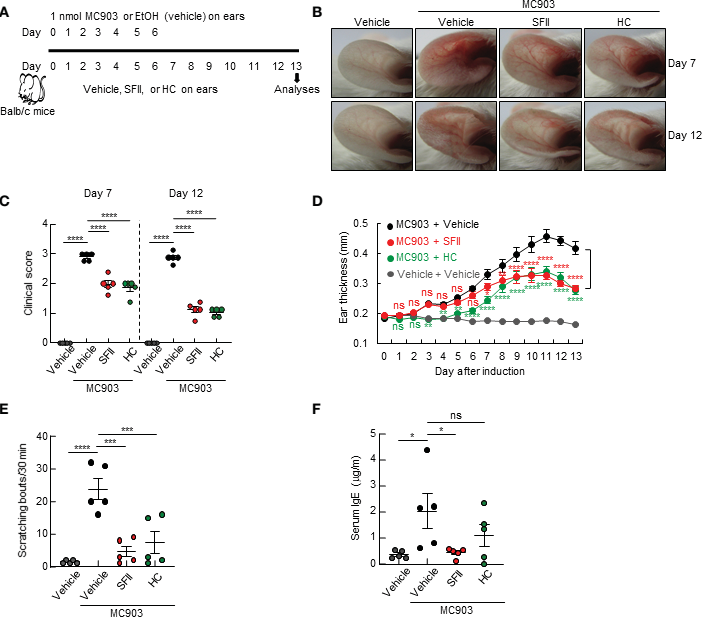
Topical SFII attenuates MC903-induced AD-like skin inflammation and pruritus **(A)** Experimental protocol of MC903-induced ear skin inflammation in BALB/c mice treated with vehicle, SFII, or HC. **(B)** MC903-induced ear skin inflammation of BALB/c mice treated with SFII, or HC on days 7 and 12. **(C)** Clinical scores of MC903-induced ear skin inflammation of BALB/c mice treated with vehicle, SFII, or HC on days 7 and 12. **(D)** Measurement of ear swelling in BALB/c mice treated with MC903 plus vehicle, SFII, or HC using a digital caliper. **(E)** Scratching bouts of BALB/c mice treated with MC903 plus vehicle, SFII, or HC on day 13. **(F)** ELISA analysis of serum IgE levels on day 13.The data are representative of the mean ± SEM of three independent experiments, with five mice per group. ns., not significant; *p < 0.05, **p < 0.01, ***p < 0.001 and ****p < 0.0001.

In this study, we examined the therapeutic efficacy of SFII on AD-like skin inflammation using an AD mouse model established by repeated topical application of MC903 on mouse skin. Mice ears were treated daily with MC903 for 7 days, followed by topical treatment with ethanol (100% EtOH, vehicle control), SFII, or HC (hydrocortisone, therapeutic control) for 13 days (**Figure 1A**). Among the vehicle-only, MC903/vehicle-, MC903/SFII-, and MC903/HC-treated groups, MC903/vehicle-treated groups developed more severe reddening and erythema of the ears compared to the other groups on days 7 and 12 (**Figures 1B, C**). In contrast, treatment with SFII attenuated skin inflammation and clinical score, which was comparable to that of HC, a widely used treatment for AD (**Figures 1B, C**). AD-like skin inflammation and ear thickening were initiated by the topical application of MC903 from day 3 (**Figure 1D**). However, treatment with SFII significantly suppressed MC903-induced ear thickening, as measured by a digital caliper from day 7, indicating the potential therapeutic effect of SFII on AD (**Figure 1D**).

### Topical SFII significantly attenuates MC903-induced pruritus and serum IgE

As pruritus and elevated levels of serum IgE are the main features of AD, we examined the effects of topical application of SFII on scratching behavior and serum IgE levels in an MC903-induced AD-like mouse model. We monitored the scratching behaviors of mice on day 13 for 30 min and found that treatment with SFII significantly suppressed MC903-induced scratching bouts, which was comparable to treatment with HC (**Figure 1E**). Total serum IgE levels on day 13 were significantly reduced in SFII-treated ears but not in HC-treated ears, which was comparable to the vehicle-only control group (**Figure 1F**). To test the dose-dependent effects of SFII on MC903-induced AD-like skin inflammation, we analyzed ear thickening, inflammation, pruritus, and total IgE levels in mouse ears treated with 0.1%, 0.5%, or 1.0% SFII. Treatment with 1.0% SFII was more effective in suppressing AD-like inflammation than treatment with 0.1% or 0.5% SFII (**Figures S1A–F**). Therefore, we evaluated the effects of 1.0% SFII on AD-like inflammation in mice.

### Topical SFII reduces MC903-induced ear thickening and immune cell infiltration

**Figure 2:**
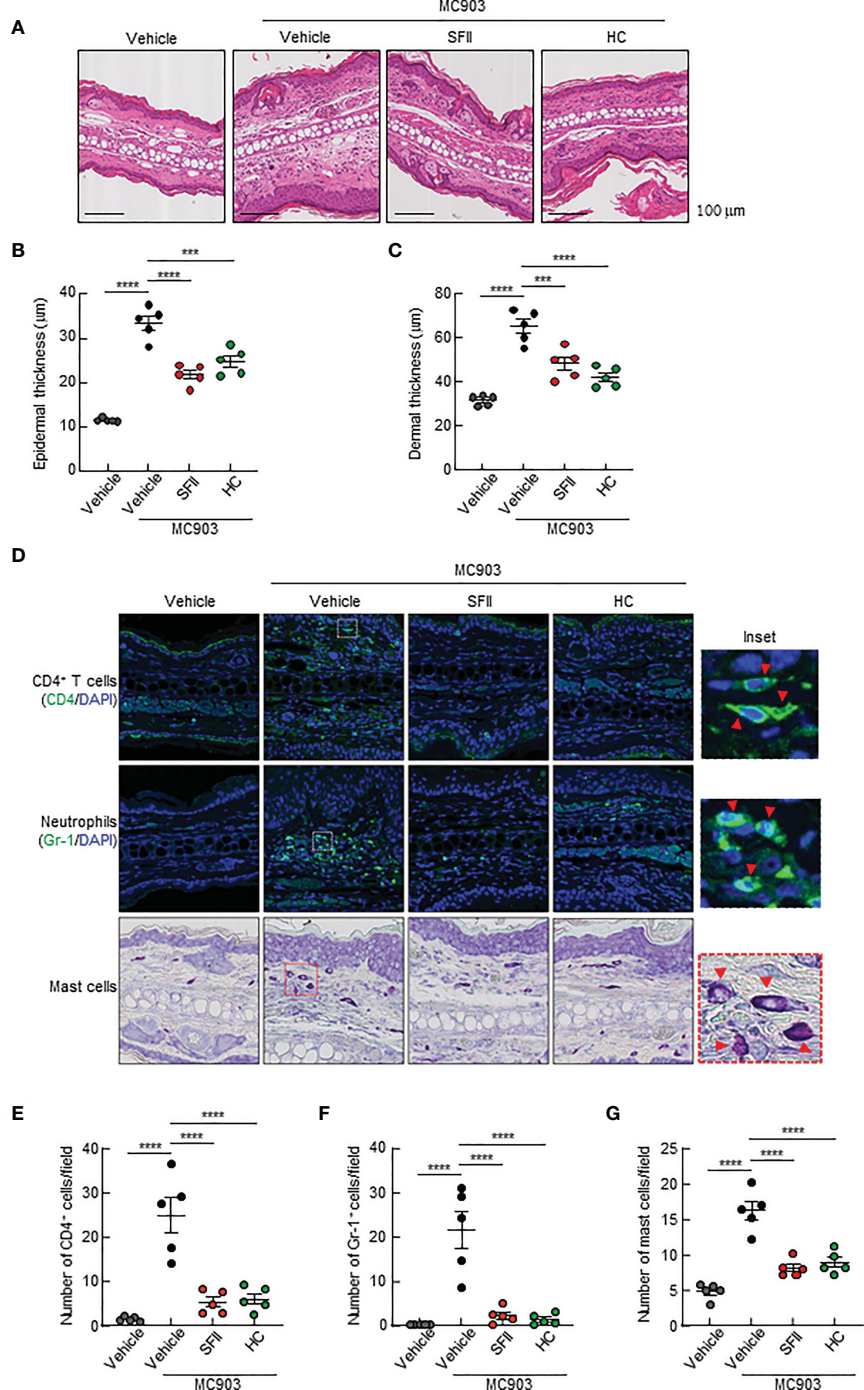
Topical SFII reduces MC903-induced ear thickening and immune cell infiltration **(A)** H&E staining of ear sections from BALB/c mice treated with vehicle, MC903 plus vehicle, SFII, or HC on day 13. **(B)** Epidermal thicknesses determined from H&E-stained ear sections. **(C)** Dermal thicknesses determined from H&E-stained ear sections. **(D)** Immunofluorescent labeling of CD4 and Gr-1, and toluidine blue staining of mast cells in ear sections on day 13. **(E)** Quantitation of CD4^+^ T cells in immunofluorescent-labeled ear sections. **(F)** Quantitation of Gr-1^+^ cells in immunofluorescent-labeled ear sections. **(G)** Quantitation of mast cells in toluidine blue-stained ear sections. All images represent five mice per group and all graphs represent three independent experiments with five mice in each group. ***p < 0.001 and ****p < 0.0001.

To determine the effects of the topical application of SFII on infiltration of immune cells, including CD4^+^ T cells, neutrophils, eosinophils, and mast cells to the site of the skin lesion, we performed H&E staining, immunofluorescence analysis, and toluidine blue staining. Histological analysis revealed that MC903/SFII-treated ears had significantly reduced epidermal and dermal thickness compared to MC903/vehicle-treated ears, which was comparable to that of HC-treated ears (**Figures 2A–C**). Infiltration of immune cells such as CD4^+^ T cells, Gr-1^+^ cells (neutrophils), mast cells, and eosinophils into lesion skin was significantly attenuated in SFII-treated ears compared to MC903/vehicle-treated ears (**Figures 2D–G**, **S2A, B**). Moreover, topical application of SFII was comparable to treatment with HC in suppressing immune cell infiltration to the skin lesions (**Figures 2D–G**, **S2A, B**).

### Topical SFII suppresses MC903-induced AD-associated cytokine production

**Figure 3:**
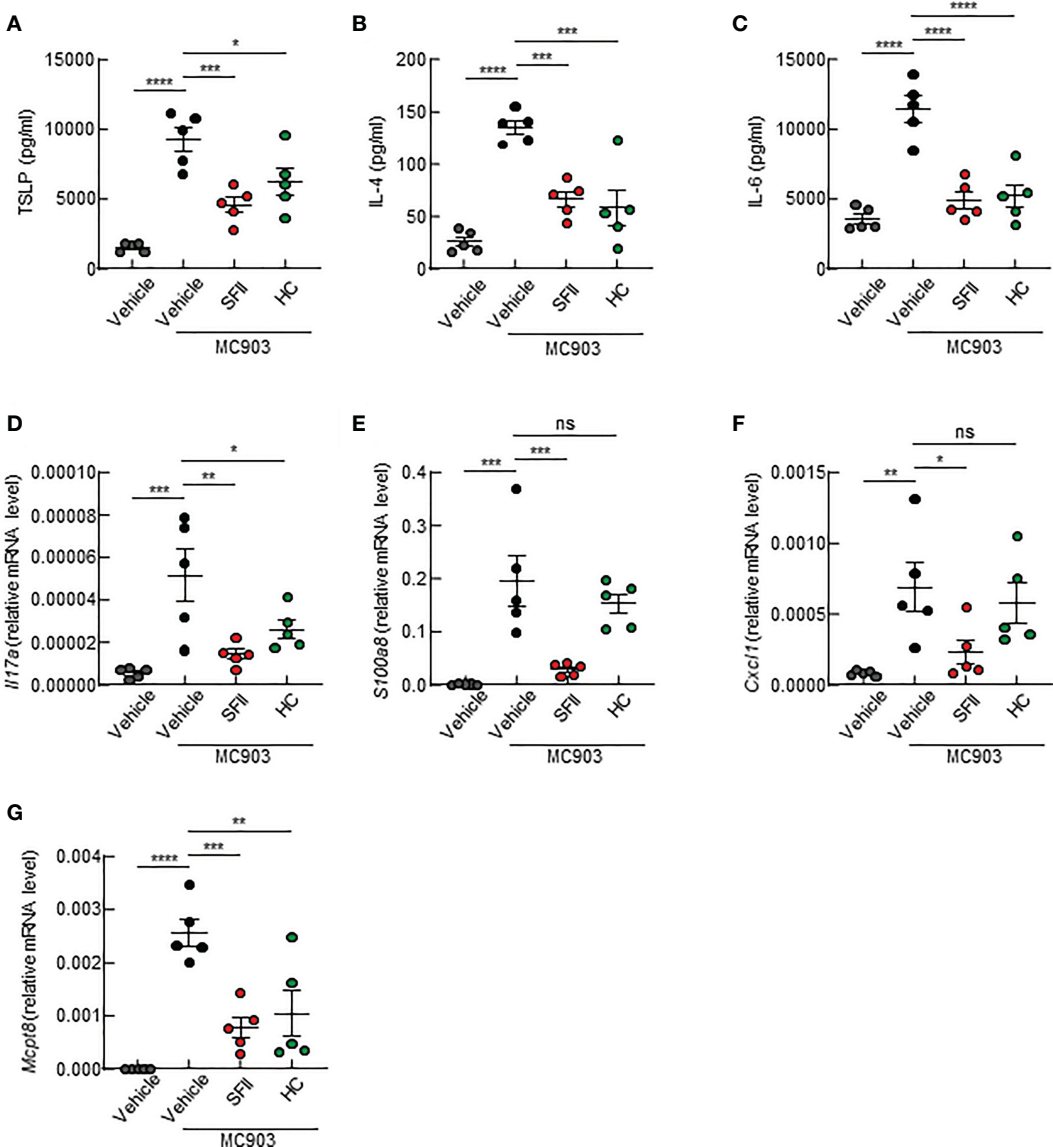
Topical SFII suppresses the production of AD-associated cytokines in ear skin induced by MC903 **(A–C)** ELISA analysis of TSLP **(A)**, IL-4 **(B)**, and IL-6 **(C)** levels in ear skin of BALB/c mice treated with MC903 plus vehicle, SFII, or HC on day 13. **(D, E)** Relative mRNA expression of *Il17a* **(D)** and *S1008a* **(E)** involved in keratinocyte proliferation on day 13. **(F)** Relative mRNA expression of *Cxcl1* involved in neutrophil recruitment on day 13. **(G)** Relative mRNA expression of *Mcpt8*, a basophil marker on day 13. All graphs represent the mean ± SEM of three independent experiments with five mice in each group. ns., not significant; *p < 0.05, **p < 0.01, ***p < 0.001 and ****p < 0.0001.

Next, we examined the effects of topical application of SFII on AD-associated cytokine production in the skin lesions of the ears. Using ELISA, we analyzed the production of Th2 cytokines, including TSLP and IL-4, which are upregulated in AD lesions. We found that topical application of SFII significantly suppressed the production of TSLP and IL-4 in ear skin compared with MC903/vehicle-treated skin (**Figures 3A, B**). The production of IL-6, which is known to be required for Th17 cell polarization, was also significantly reduced in MC903/SFII-treated skin compared to that in MC903/vehicle-treated skin (**Figure 3C**). High levels of IL-17A (Th17 cytokine) and S100a8 (a Th17/22-related product) have also been reported in patients with AD (37, 38). Q-PCR analysis showed that the mRNA expression levels of *Il17a* and *S100a8* in MC903/SFII-treated skin were significantly lower than those in MC903/vehicle-treated skin (**Figures 3D, E**). Interestingly, treatment with SFII was more effective in suppressing the expression of *Il17a* and *S100a8* than treatment with HC (**Figures 3D, E**). Treatment with SFII also significantly suppressed the mRNA expression of *Cxcl1* (a neutrophil chemoattractant) and *Mcpt8* (a basophil marker) (**Figures 3F, G**). MC903-induced *Ccl7* expression (an eosinophil chemoattractant) was significantly inhibited by SFII (**Figure S2E**). However, treatment with MC903 had no effect on the expression of other eosinophil chemoattractants such as *Il5*, *Ccl11*, and *Ccl17* nor on the production of IL-5 in the lesioned skin (**Figures S2C, D, F, G**).

### SFII significantly inhibits TSLP production from human primary keratinocytes *in vitro*

**Figure 4:**
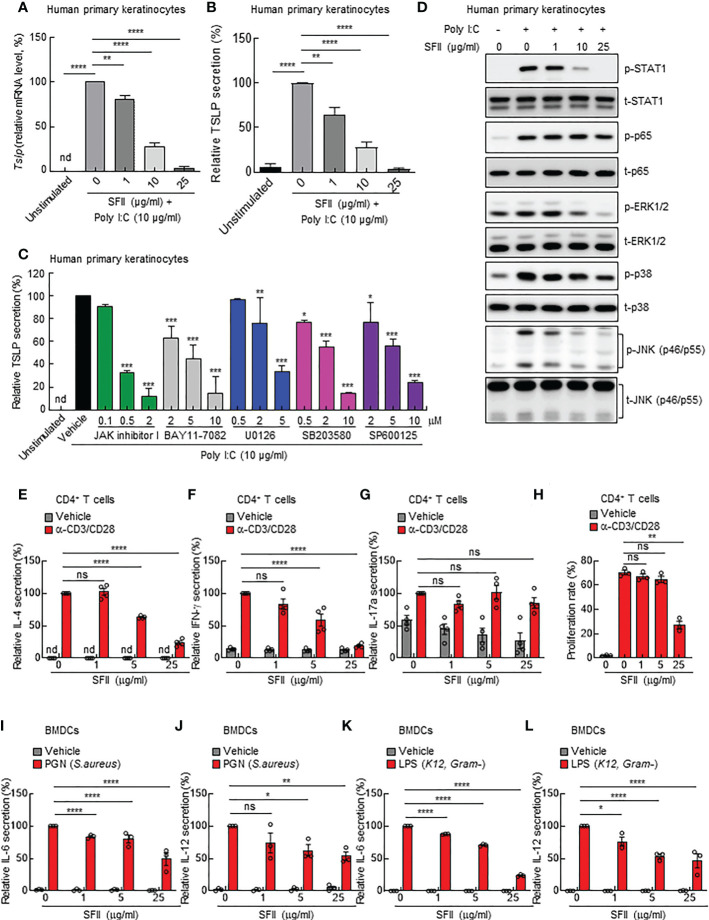
SFII directly inhibits AD-associated cytokine production from human primary keratinocytes, mouse CD4^+^ T cells, and mouse BMDCs *in vitro* **(A)** Relative mRNA expression of TSLP in human primary keratinocytes stimulated with poly(I:C) plus vehicle or different doses of SFII for 6 h. **(B)** ELISA analysis of TSLP production in culture supernatant of human primary keratinocytes stimulated with poly(I:C) plus vehicle or different doses of SFII for 24 h. **(C)** ELISA analysis of TSLP production in culture supernatants of human primary keratinocytes stimulated with poly(I:C) plus vehicle or the indicated inhibitors at different doses. **(D)** Immunoblotting analysis of the expression levels of p-STAT1, t-STAT-1, p-p65, t-p65, p-ERK, t-ERK, p-p38, t-p38, p-JNK, and t-JNK in the total protein from human primary keratinocytes stimulated with poly(I:C) plus vehicle or different doses of SFII for 3 h. **(E–G)** ELISA analysis of IL-4 **(E)**, IFN-γ **(F)** and IL-17A **(G)** production in culture supernatants of mouse CD4^+^ T cells stimulated with anti-CD3 (5 μg/ml) and anti-CD28 (2 μg/ml) antibodies plus vehicle or different doses of SFII for 72 h. **(H)** Quantification of CFSE analysis of mouse CD4^+^ T cells stimulated with anti-CD3 (5 μg/ml) and anti-CD28 (2 μg/ml) antibodies plus vehicle or different doses of SFII for 72 h. **(I, J)** ELISA analysis of IL-6 **(I)** and IL-12 **(J)** production in mouse BMDC culture supernatants stimulated with PGN plus vehicle or different doses of SFII for 24 h. **(K, L)** ELISA analysis of IL-6 **(K)** and IL-12 **(L)** production in mouse BMDC culture supernatant stimulated with LPS plus vehicle or different doses of SFII for 24 h. All graphs represent the mean ± SEM from three or four independent experiments. nd., not detected; ns., not significant; *p < 0.05, **p < 0.01, ***p < 0.001 and ****p < 0.0001.

As we observed significantly attenuated TSLP production in topical SFII-treated mouse skin (**Figure 3A**), we examined the effects of SFII on poly(I:C)-induced TSLP production in human primary keratinocytes *in vitro*. TSLP levels in both the cell lysate and cell culture supernatants were determined using Q-PCR analysis and ELISA, respectively. The data showed that treatment with SFII significantly and dose-dependently attenuated the mRNA and protein levels of TSLP induced by poly (I:C) (**Figures 4A, B**). To assess the molecular mechanisms by which poly(I:C) induces TSLP expression, we analyzed poly (I:C)-induced TSLP production in the presence or absence of the indicated inhibitors, including JAK inhibitor I, BAY11-7082 (a NF-κB inhibitor), U0126 (a MEK inhibitor), SB203580 (a p38 MAPK inhibitor), or SP600125 (a JNK inhibitor) (**Figure 4C**). The ELISA data showed that TSLP production was significantly and dose-dependently suppressed by the JAK I inhibitors, BAY11-7082, U0126, SB203580, and SP600125, indicating that TLSP production is dependent on STAT1, NF-κB, ERK1/2, p38 MAPK, and JNK signaling pathways (**Figure 4C**). Additionally, we identified the effect of SFII on the activation of these signaling molecules involved in Poly(I:C)-induced TSLP production and found that high doses of SFII markedly and significantly inhibited Poly(I:C)-induced phosphorylation of STAT1, ERK1/2, p38 MAPK, and JNK (**Figure 4D**, **S3A, C–F**). However, the poly (I:C)-induced phosphorylation of p65 was slightly reduced by a high dose of SFII (**Figures 4D**, **S3B**).

### SFII directly inhibits AD-associated cytokine production in mouse BMDCs and CD4^+^ T cells *in vitro*

Furthermore, we aimed to determine whether SFII could directly inhibit the activation of immune cells or whether reduced TSLP production by SFII causes attenuated activation of inflammatory immune cells *in vivo*. Because CD4^+^ T cells are mainly involved in MC903-induced AD-like skin inflammation, we isolated CD4^+^ T cells from the mouse spleen and stimulated them with anti-CD3/CD28 antibodies for 3 days in the presence of different concentrations of SFII. The ELISA data showed that treatment with SFII significantly and dose-dependently suppressed the production of IL-4 and IFN-γ induced by anti-CD3/CD28 antibodies (**Figures 4E, F**). These data suggest that SFII contributes to the attenuation of AD-like inflammation by directly blocking the production of IL-4 and IFN-γ by CD4^+^ T cells. However, the production of IL-17Ainduced by anti-CD3/CD28 antibodies was not affected by SFII treatment (**Figure 4G**), suggesting that SFII-induced decline in IL-17A production in mouse skin may be caused by secondary effects. In addition, carboxyfluorescein diacetate succinimidyl ester (CFSE) cell division analysis showed that treatment with SFII significantly and dose-dependently prevented CD4^+^ T cell proliferation (**Figure 4H**).

Since dendritic cells (DCs) are mainly involved in MC903-induced AD-like skin inflammation, we determined the effect of SFII on BMDC activation. We generated mouse BMDCs in the presence of GM-CSF for 6 days and then stimulated BMDCs with the TLR agonists PGN (*S. aureus*) or LPS (*K12*) in the presence of different concentrations of SFII. The ELISA data revealed that treatment with SFII significantly and dose-dependently attenuated IL-6 and IL-12 production induced by PGN or LPS in BMDCs (**Figures 4I–L**), indicating that treatment with SFII also directly suppresses the activation of dendritic cells.

### Topical SFII is more effective at suppressing serum IgE, IL-4, and TSLP production than topical baicalein

**Figure 5:**
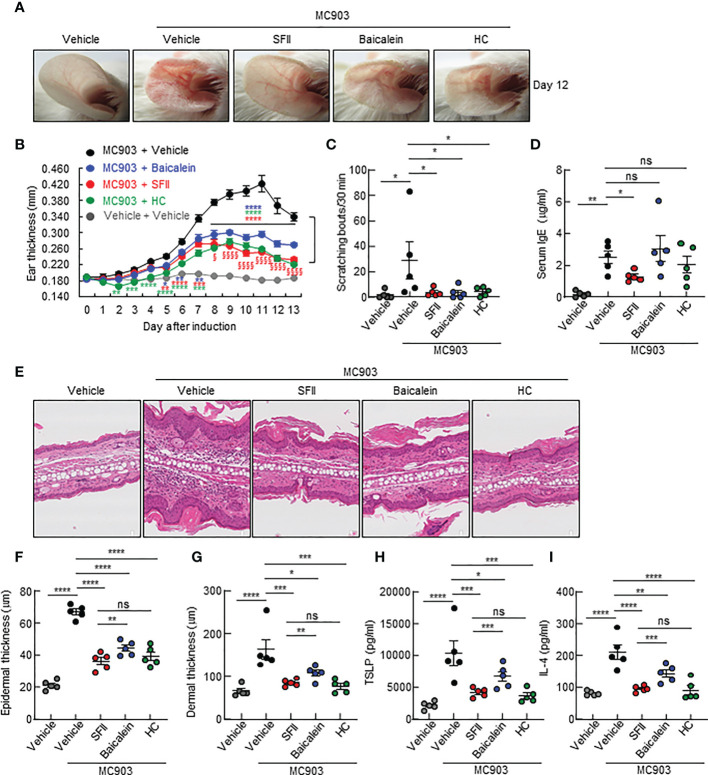
Topical SFII is more effective in MC903-induced suppression of skin thickening and the production of serum IgE, IL-4, and TSLP compared to baicalein **(A)** MC903-induced ear skin inflammation of BALB/c mice treated with vehicle, SFII, Baicalein, or HC on day 12. **(B)** Ear swelling measurements by a digital caliper of BALB/c mice treated with MC903 plus vehicle, SFII, baicalein, or HC. *p < 0.05, **p < 0.01, ***p < 0.001 and ****p < 0.0001 vs MC903/vehicle. ^§^p < 0.05 and ^§§§§^p < 0.0001 vs baicalein. **(C)** Scratching bouts of BALB/c mice treated with MC903 plus vehicle, SFII, baicalein, or HC on day 13. **(D)** ELISA analysis of serum IgE levels on day 13. **(E)** H&E staining of ear sections from BALB/c mice treated with MC903 plus vehicle, SFII, baicalein, or HC on day 13. **(F)** Epidermal thickness determined from H&E-stained ear sections. **(G)** Dermal thickness determined from H&E-stained ear sections. **(H, I)** ELISA analysis of TSLP **(H)** and IL-4 **(I)** in ear skin of BALB/c mice treated with MC903 plus vehicle, SFII, baicalein, or HC on day 13. All images represent five mice per group, and all graphs represent the mean ± SEM from two independent experiments, with five mice in each group. ns., not significant; *p < 0.05, **p < 0.01, ***p < 0.001 and ****p < 0.0001.

**Figure 6:**
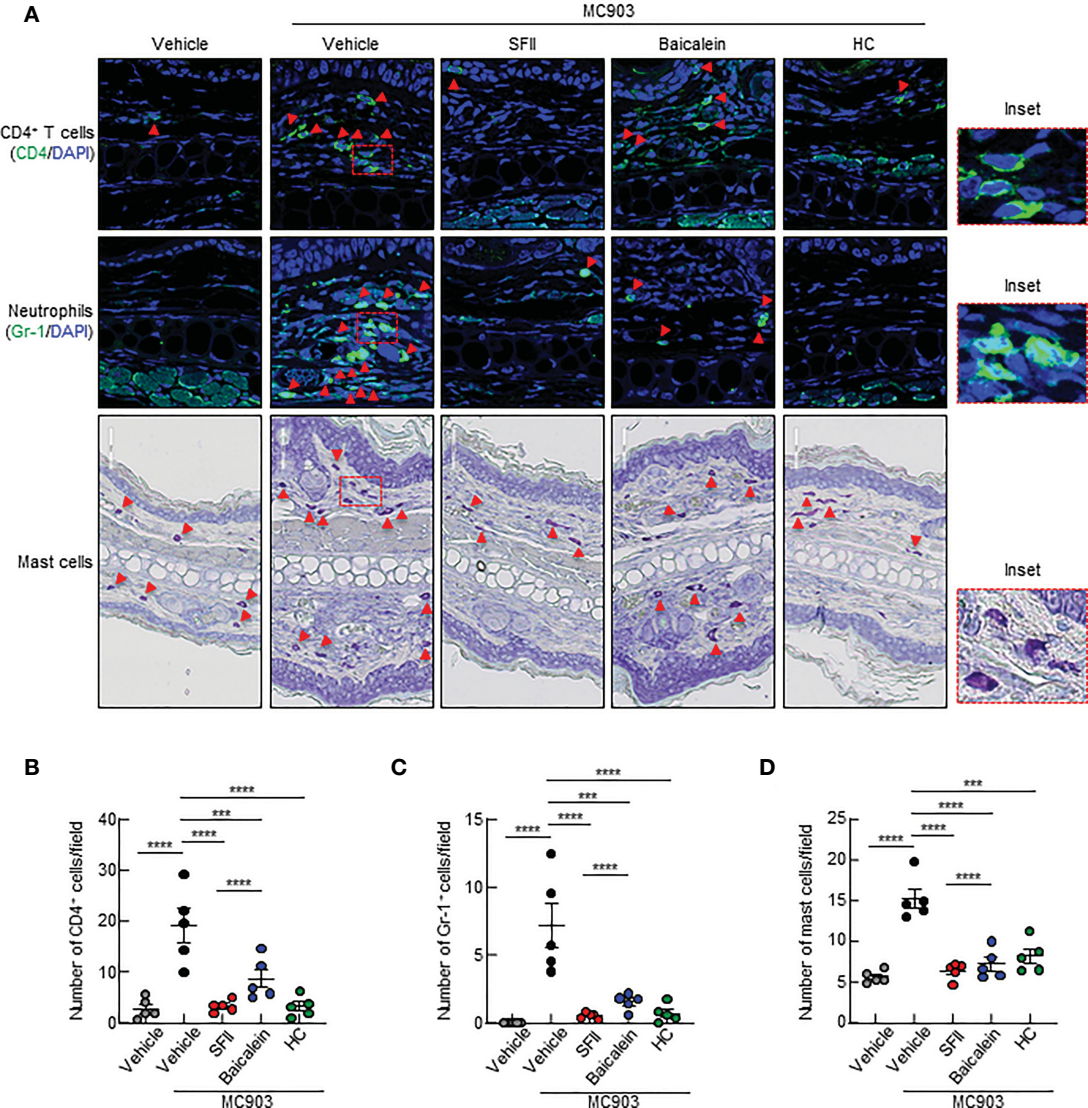
Topical SFII is more effective at suppressing MC903-induced infiltration of CD4^+^ T cells and neutrophils into skin compared to baicalein **(A)** Immunofluorescent labeling of CD4 and Gr-1, and toluidine blue staining of mast cells in ear sections on day 13. **(B)** Quantitation of CD4^+^ T cells in immunofluorescent-labeled ear sections. **(C)** Quantitation of Gr-1^+^ cells in immunofluorescent-labeled ear sections. **(D)** Quantitation of mast cells in toluidine blue-stained ear sections. All images represent five mice per group, and all graphs are representative of the mean ± SEM from two independent experiments, with five mice in each group. ns., not significant; ***p < 0.001 and ****p < 0.0001.

A previous study showed that topical baicalein, a compound from *Scutellaria baicalensis*, significantly reduces immune cell infiltration and skin thickening in the AD tissues of NC/Nga mice (30). In addition, intraperitoneal or oral administration of baicalein has been shown to inhibit histamine- or compound 48/80-induced scratching behavior in mice (39, 40). We compared the therapeutic efficacy of SFII (1.0%) and baicalein (0.72%) at the same molar concentration (26.7 mM) in MC903-induced AD-like skin inflammation. Topical treatment with SFII or baicalein suppressed reddening, erythema, and swelling on day 13 (**Figures 5A, B**). Scratching bouts were also significantly suppressed in both SFII- and baicalein-treated mice with comparable effects (**Figure 5C**). However, topical treatment with SFII was more effective in suppressing serum IgE levels (**Figure 5D**) and production of TSLP and IL-4 (**Figures 5H, I**) compared to topical treatment with baicalein. Histological analysis revealed that topical treatment with SFII and baicalein reduced epidermal and dermal thickness compared to the MC903/vehicle-treated group (**Figures 5E–G**). However, topical treatment with SFII more effectively suppressed epidermal and dermal thickness than treatment with baicalein (**Figures 5E–G**). Moreover, immunofluorescence analysis and toluidine blue staining showed that infiltration of CD4^+^ T cells, Gr-1^+^ cells (neutrophils), and mast cells into lesion skin was significantly attenuated in SFII-, baicalein-, or HC-treated ears compared to MC903/vehicle-treated ears (**Figures 6A–D**). However, topical treatment with SFII more effectively suppressed infiltrating CD4^+^ T cells, Gr-1^+^ cells (neutrophils), and mast cells than baicalein (**Figures 6A–D**). The topical application of SFII was comparable to treatment with HC in suppressing immune cell infiltration into the skin lesions (**Figures 6A–D**).

## Discussion

To date, topical glucocorticoids and the topical calcineurin inhibitor, tacrolimus, have been used as first-line therapies to treat patients with moderate-to-severe forms of AD (41, 42). However, the long-term use of immunosuppressive agents and glucocorticoids has been reported to cause multiple side effects (42–45). Therefore, the discovery of safe and effective therapeutics against AD is essential. Here, we showed that SFII, a flavonoid derived from *Scutellaria baicalensis*, is a potential therapeutic target for the treatment of AD. Our findings show that topical SFII inhibits MC903-induced erythema, edema, swelling, AD-associated skin inflammation, and pruritus in mice, similar to topical HC, a corticosteroid. Natural products and flavonoids are known to exhibit powerful antioxidants and potential pharmacological effects in allergic diseases, such as asthma, AD, anaphylaxis, and food allergy, and substantial scientific evidence on their safety profile and effectiveness exists (46–49). Thus, the discovery of natural products-based drugs could be one of the approaches for the treatment of AD in humans (50).

We observed a significant reduction in MC903-induced epidermal and dermal thickening following topical SFII use, which is comparable to that observed with topical HC or tacrolimus. Concomitantly, we observed that topical SFII significantly reduced MC903-induced S100a8 expression in the skin lesions. The exact mechanism by which MC903 induces S100a8 expression is still unknown; however, induction of S100a8 expression is mediated by IL-17A through the p38 MAPK pathway (51) and MC903 also triggers IL-17A production. S100A8 is known to promote keratinocyte proliferation (52) and its expression is markedly increased in acute and chronic AD skin (53). Our data suggest that reduced S100a8 expression by SFII may contribute to decreased epidermal thickening. Skin dermal thickening and allergic inflammation are promoted by immune cell infiltration. Of these, effector CD4^+^ T cells and mast cells are crucial for the pathogenesis of AD by producing Th2 cytokines, such as IL-4 and IL-13 (53). Here, we revealed that topical SFII significantly inhibited the infiltration of CD4^+^ T cells and mast cells, as well as IL-4 production in skin lesions induced by MC903, which was comparable to that induced by topical HC. Skin-infiltrating neutrophils and basophils also promote AD inflammation and itch (9, 54). Epithelial cell-derived CXCL1 is crucial in recruiting and activating neutrophils (9, 55). We further showed that topical SFII significantly suppressed the expression of the neutrophil chemoattractant, *Cxcl1*, and neutrophil infiltration in skin lesions. The mechanism by which CXCL1 expression is induced by MC903 remains unclear. However, protease-activated receptor agonists or IL-17A increases CXCL1 production in keratinocytes (9, 56) and CXCL1 expression is unaffected by the loss of TSLPR or neutrophil depletion (9). In addition, we observed that topical SFII reduced *Mcpt8* expression, a specific marker for murine basophils, suggesting reduced infiltration of basophils in the lesioned skin. We also showed that topical SFII reduced eosinophil recruitment and *Ccl7* expression in the lesioned skin. Eosinophils are associated with disease severity in patients with AD (57) and involved in itch, dermal thickening, and water loss in AD-like mouse models (58, 59). Although our data showed that the expression of eosinophil-associated factors (IL-5, *Ccl11*, and *Ccl17*) did not change following MC903 treatment for 7 consecutive days (additional 6 days without MC903), other studies have shown that the expression of IL-5 and CCL24 (eotaxin-2) are significantly increased from day 9 or on day 14 after MC903 treatment (58, 60). These findings suggest that the expression of eosinophil-associated factors in lesioned skin appears to be a late immune response. Thus, our findings suggest that reduced skin-infiltrating CD4^+^ T cells, mast cells, neutrophils, basophils, and eosinophils may cause decreased dermal thickening and ameliorate allergic inflammatory responses.

A recent study has shown that systemic depletion of neutrophils using an anti-Gr-1 antibody dramatically inhibits scratching behavior in an MC903-induced AD model, suggesting that neutrophils mediate itching (9). In parallel, we showed that topical SFII significantly suppressed MC903-induced scratching bouts, which were comparable to that of topical HC. Moreover, CXCL1 and IL-4 have been shown to directly stimulate itch-sensory neurons, leading to chronic itching (21) (61, 62). Thus, our data suggest that reduced IL-4 and CXCL1 production and reduced neutrophil infiltration following SFII administration may contribute to the inhibition of the itch response.

Epithelial cell-derived TSLP has been shown to trigger Th2 immune responses and skin-infiltrating immune cells, including dendritic cells, basophils, CD4^+^ T cells, and mast cells (18). TSLP is also a pruritogen that activates neurons to induce itch (63). We observed that topical SFII significantly inhibited MC903-induced TSLP production in the skin lesions. Moreover, our *in vitro* studies showed that SFII directly suppressed TSLP production by human primary keratinocytes *via* inhibition of STAT1, ERK1/2, p38 MAPK, and JNK signaling pathways, indicating that decreased TSLP by SFII may result in reduced Th2 inflammation and infiltrated immune cells as well as the suppression of itching.

Furthermore, our *in vitro* studies of BMDCs revealed that SFII significantly suppressed the production of IL-6 and IL-12 induced by the TLR ligands PGN or LPS. In addition, we showed that SFII significantly suppressed the production of IL-4 and IFN-γ in anti-CD3/CD28-stimulated CD4^+^ T cells from the mouse spleen and T cell proliferation, indicating that SFII directly suppresses immune cell activation. Overall, our findings suggest that reduced TSLP production in keratinocytes by SFII may result in reduced Th2 inflammatory responses and that SFII could directly regulate the activation of immune cells, including DCs, Th2, and Th1 cells. *In vitro* IL-17A production in anti-CD3/CD28-stimulated CD4^+^ T cells was not affected by SFII, whereas topical SFII significantly inhibited IL-17A expression in MC903-induced skin lesions *in vivo*, suggesting that SFII may indirectly suppress IL-17A production in mouse skin. Thus, one possible explanation is that reduced IL-6 and IL-12 production by SFII, which is involved in Th17 polarization (64, 65), may result in reduced IL-17A production *in vivo*.

Lastly, we showed that topical SFII and baicalein significantly suppressed MC903-induced skin thickening, pruritus, and AD-related cytokine production compared to the MC903/vehicle-treated group. However, topical SFII is more effective in suppressing MC903-induced skin thickening and IL-4 and TSLP production in lesioned skin compared to topical baicalein. Interestingly, we observed that MC903-induced serum IgE levels were significantly suppressed by topical SFII alone, but not by topical baicalein or HC. A previous study has shown that intraperitoneal injection of baicalein reduces OVA-induced serum IgE levels in a mouse model of airway inflammation (66). Thus, these contrasting findings are likely due to differences in disease models, drug administration methods, and duration of treatment. Enhanced total IgE levels by HC have been reported previously (67, 68), and consistent with this, we confirmed that topical HC does not significantly reduce serum IgE levels in the MC903-induced AD-like mouse model. Further studies are required to understand the mechanism by which SFII effectively suppresses IgE production. Furthermore, we showed that MC903-induced infiltration of immune cells, including CD4^+^ T cells, neutrophils, and mast cells, was significantly reduced in skin treated with topical SFII, baicalein, and HC-treated skin. However, topical SFII was more effective than baicalein at suppressing the infiltration of immune cells, including CD4^+^ T cells and neutrophils, but not mast cells. Overall, our findings suggest that topical SFII has a more beneficial effect on AD treatment than topical baicalein.

In conclusion, we show that topical SFII can ameliorate AD-like pathology by regulating multiple targets in the MC903 mouse model. Moreover, we revealed that topical SFII has a beneficial effect on the suppression of IgE production compared to topical baicalein or HC. Therefore, our findings suggest the effectiveness of SFII in the pharmacological management and treatment of complex AD and pruritus as a single agent therapy or combination therapy with corticosteroids. Further studies are needed to determine the specific targets of SFII in cells, and clinical studies are warranted to determine the effects of SFII in patients with AD.

## Data availability statement

The original contributions presented in the study are included in the article/**Supplementary Material**. Further inquiries can be directed to the corresponding author.

## Ethics statement

The animal study was reviewed and approved by Seoul National University Hospital Institutional Animal Care and Use Committee.

## Author contributions

YL and J-HO designed and performed experiments, analyzed the data, and prepared the figures. NL and H-JJ performed experiments and prepared research reagents. K-SA, S-RO, DL, and JC conceived the project and supervised this research. YL and JC wrote this original draft. All authors contributed to the article and approved the submitted version.

## Supplementary material

Supplementary Figure 1 Topical 1.0% SFII is more effective at attenuating MC903-induced AD-like skin inflammation and pruritus compared to 0.1% and 0.5% SFII **(A)** MC903-induced ear skin inflammation of BALB/c mice treated with 0.1%, 0.5%, or 1.0% SFII, tacrolimus, or HC on day 12. **(B)** Measurement of ear swelling in BALB/c mice treated with MC903 plus vehicle, 0.1%, 0.5%, or 1.0% SFII, tacrolimus, or HC using a digital caliper. **(C)** Scratching bouts of BALB/c mice treated with MC903 plus vehicle, 0.1%, 0.5%, or 1.0% SFII, tacrolimus, or HC on day 13. **(D)** ELISA analysis of serum IgE levels on day 13. **(E)** H&E staining of ear sections from BALB/c mice treated with vehicle, MC903 plus vehicle, 0.1%, 0.5%, or 1.0% SFII, tacrolimus, or HC on day 13. **(F)** Epidermal and dermal thickness determined from H&E-stained ear sections. The data are representative of the mean ± SEM of two independent experiments, with five mice per group. ns., not significant; * p < 0.05, ** p < 0.01, *** p < 0.001 and **** p < 0.0001.

Supplementary Figure 2 Topical SFII reduces MC903-induced eosinophil infiltration and Ccl7 expression **(A)** Immunofluorescent labeling of eosinophils in ear sections on day 13. **(B)** Quantitation of eosinophils in immunofluorescent-labeled ear sections. **(C)** ELISA analysis of IL-5 levels in ear skin of BALB/c mice treated with MC903 plus vehicle, SFII, or HC on day 13. **(D–G)** Relative mRNA expression of *Il5* **(D)**, *Ccl7* **(E)**, *Ccl11* **(F)**, and *Ccl17* **(G)** involved in eosinophil-associated factors on day 13. All images represent five mice per group, and all graphs are representative of the mean ± SEM from three independent experiments, with five mice in each group. ns., not significant; ** p < 0.01, *** p < 0.001 and **** p < 0.0001.

Supplementary Figure 3 SFII markedly and significantly inhibits Poly(I:C)-induced phosphorylation of STAT1, ERK1/2, p38 MAPK, and JNK **(A)** Quantitation of p-STAT-1/t-STAT-1 in western blot data. **(B)** Quantitation of p-p65/t-p65 in western blot data. **(C)** Quantitation of p-ERK1/2/t-ERK1/2 in western blot data. **(D)** Quantitation of p-p38/t-p38 in western blot data. **(E, F)** Quantitation of p-JNK/t-JNK (55 kDa) **(E)** and p-JNK/t-JNK (46 kDa) **(F)** in western blot data. All graphs represent the mean ± SEM from four independent experiments. ns., not significant; * p < 0.05, ** p < 0.01 and *** p < 0.001.
