# Corrigendum: Topical Skullcapflavone II attenuates atopic dermatitis in a mouse model by directly inhibiting associated cytokines in different cell types

**DOI:** 10.3389/fimmu.2023.1150830

**Published:** 2023-02-07

**Authors:** Youngae Lee, Jang-Hee Oh, Na Li, Hyun-Jae Jang, Kyung-Seop Ahn, Sei-Ryang Oh, Dong Hun Lee, Jin Ho Chung

**Affiliations:** ^1^ Department of Dermatology, Seoul National University College of Medicine, Seoul, Republic of Korea; ^2^ Institute of Human-Environment Interface Biology, Medical Research Center, Seoul National University, Seoul, Republic of Korea; ^3^ Laboratory of Cutaneous Aging Research, Biomedical Research Institute, Seoul National University Hospital, Seoul, Republic of Korea; ^4^ Department of Biomedical Sciences, Seoul National University Graduate School, Seoul, Republic of Korea; ^5^ Natural Medicine Research Center, Korea Research Institute of Bioscience and Biotechnology, Cheong-ju, Chungcheongbuk-do, Republic of Korea

**Keywords:** Skullcapflavone II, atopic dermatitis, pruritus, Th2 cytokines, IgE

In the published article, there was an error in the author list, and authors Youngae Lee and Jang-Hee Oh were erroneously not annotated as co-first authors. The corrected author list appears below.

Youngae Lee^1,2,3†^, Jang-Hee Oh^1,2,3†^, Na Li^1,2,3,4^, Hyun-Jae Jang^5^, Kyung-Seop Ahn^5^, Sei-Ryang Oh^5^, Dong Hun Lee^1,2,3^ and Jin Ho Chung^1,2,3,4*^

*^†^ These authors have contributed equally to this work.*

The authors apologize for this error and state that this does not change the scientific conclusions of the article in any way. The original article has been updated.

