# Supplementary figures and images for: Topical Skullcapflavone II attenuates atopic dermatitis in a mouse model by directly inhibiting associated cytokines in different cell types
Source: Front Immunol. 2022 Dec 20;13:1064515. doi: 10.3389/fimmu.2022.1064515 (PMC9808403; doi:10.3389/fimmu.2022.1064515)

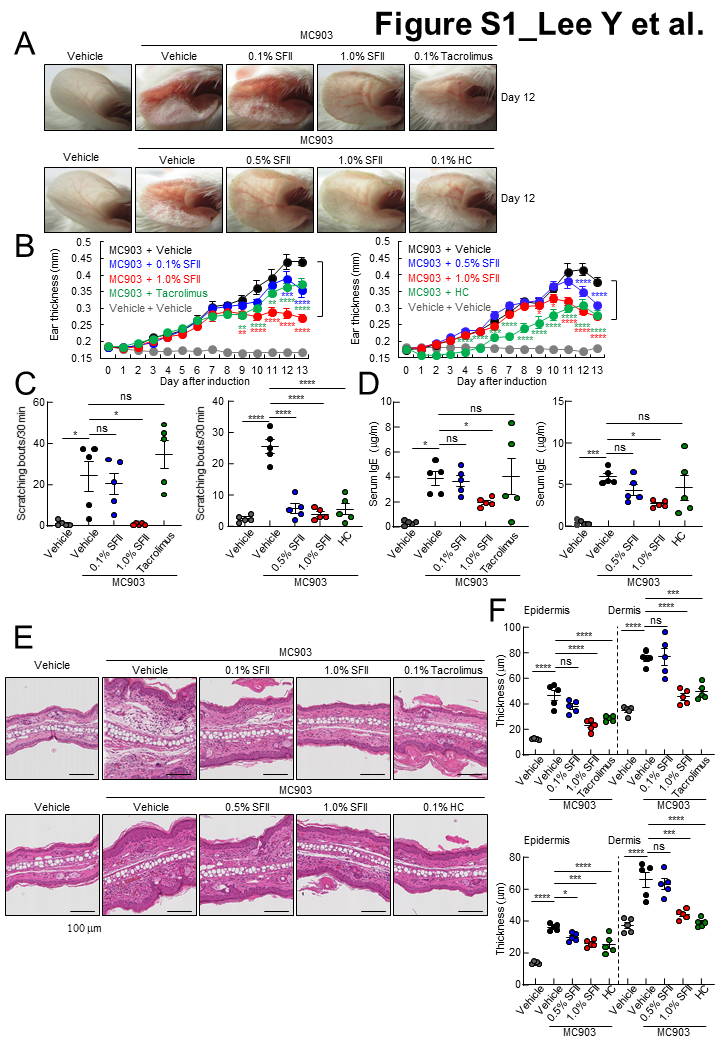

Supplement: Supplementary Figure 1 — Topical 1.0% SFII is more effective at attenuating MC903-induced AD-like skin inflammation and pruritus compared to 0.1% and 0.5% SFII (A) MC903-induced ear skin inflammation of BALB/c mice treated with 0.1%, 0.5%, or 1.0% SFII, tacrolimus, or HC on day 12. (B) Measurement of ear swelling in BALB/c mice treated with MC903 plus vehicle, 0.1%, 0.5%, or 1.0% SFII, tacrolimus, or HC using a digital caliper. (C) Scratching bouts of BALB/c mice treated with MC903 plus vehicle, 0.1%, 0.5%, or 1.0% SFII, tacrolimus, or HC on day 13. (D) ELISA analysis of serum IgE levels on day 13. (E) H&E staining of ear sections from BALB/c mice treated with vehicle, MC903 plus vehicle, 0.1%, 0.5%, or 1.0% SFII, tacrolimus, or HC on day 13. (F) Epidermal and dermal thickness determined from H&E-stained ear sections. The data are representative of the mean ± SEM of two independent experiments, with five mice per group. ns., not significant; * p < 0.05, ** p < 0.01, *** p < 0.001 and **** p < 0.0001. [file Image_1.tif]

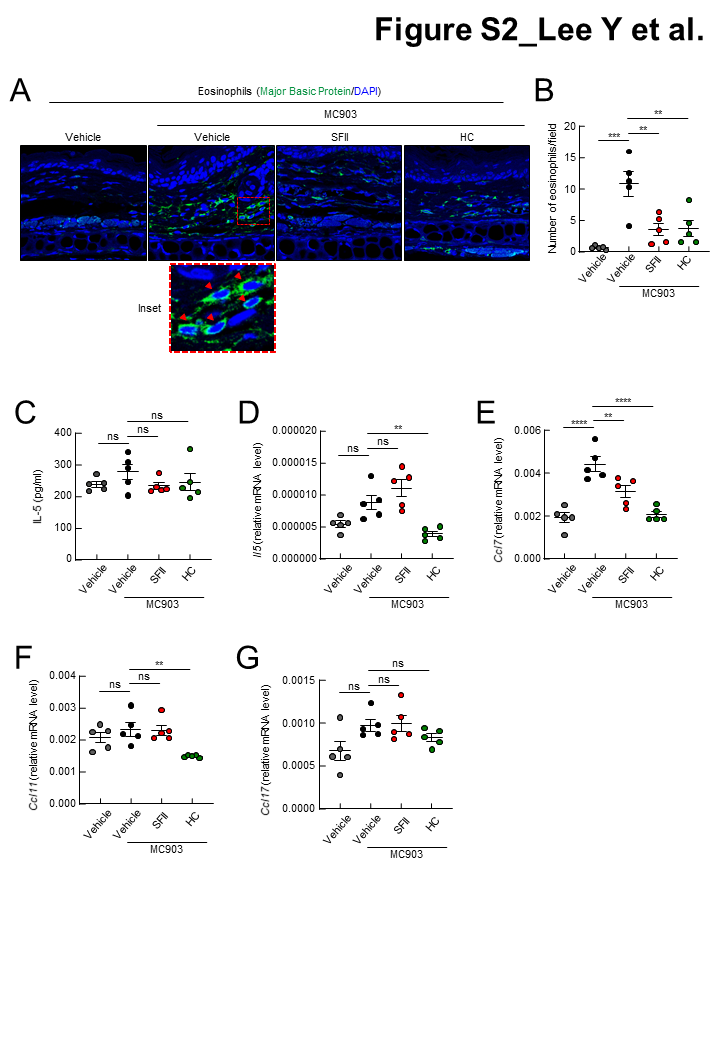

Supplement: Supplementary Figure 2 — Topical SFII reduces MC903-induced eosinophil infiltration and Ccl7 expression (A) Immunofluorescent labeling of eosinophils in ear sections on day 13. (B) Quantitation of eosinophils in immunofluorescent-labeled ear sections. (C) ELISA analysis of IL-5 levels in ear skin of BALB/c mice treated with MC903 plus vehicle, SFII, or HC on day 13. (D–G) Relative mRNA expression of Il5 (D), Ccl7 (E), Ccl11 (F), and Ccl17 (G) involved in eosinophil-associated factors on day 13. All images represent five mice per group, and all graphs are representative of the mean ± SEM from three independent experiments, with five mice in each group. ns., not significant; ** p < 0.01, *** p < 0.001 and **** p < 0.0001. [file Image_2.tif]

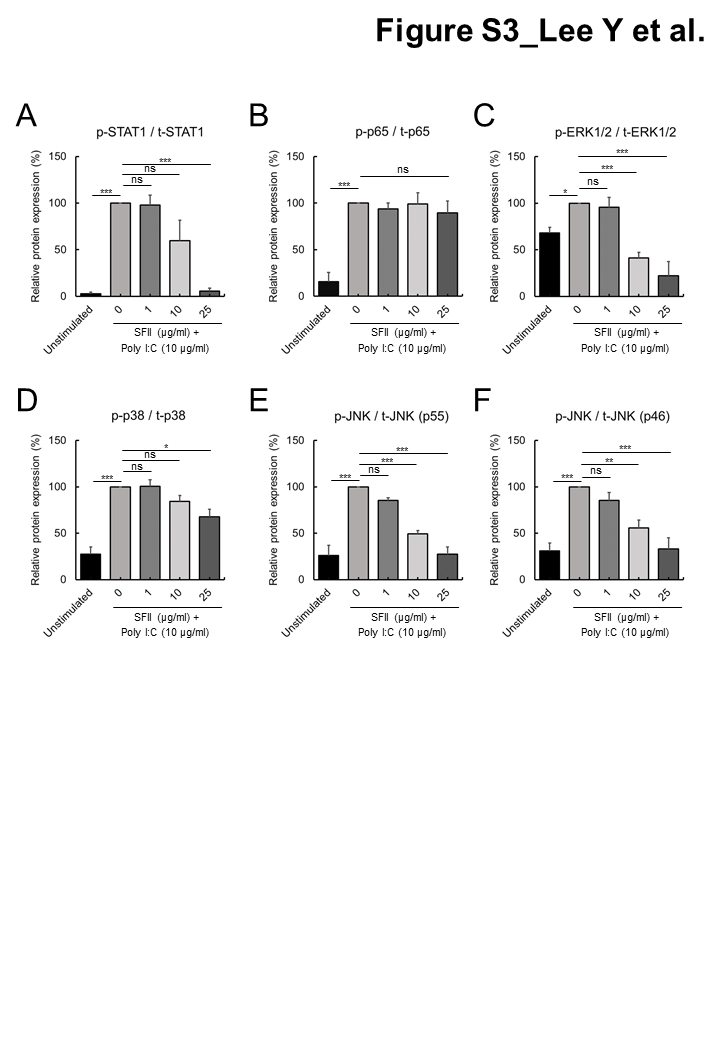

Supplement: Supplementary Figure 3 — SFII markedly and significantly inhibits Poly(I:C)-induced phosphorylation of STAT1, ERK1/2, p38 MAPK, and JNK (A) Quantitation of p-STAT-1/t-STAT-1 in western blot data. (B) Quantitation of p-p65/t-p65 in western blot data. (C) Quantitation of p-ERK1/2/t-ERK1/2 in western blot data. (D) Quantitation of p-p38/t-p38 in western blot data. (E, F) Quantitation of p-JNK/t-JNK (55 kDa) (E) and p-JNK/t-JNK (46 kDa) (F) in western blot data. All graphs represent the mean ± SEM from four independent experiments. ns., not significant; * p < 0.05, ** p < 0.01 and *** p < 0.001. [file Image_3.tif]
